# Hepatectomy for Hepatocellular Carcinoma Complicated by Vasculitis Flare

**DOI:** 10.1155/2010/841754

**Published:** 2010-09-01

**Authors:** Zeinab Abdi, Svetlana Krasnokutsky, Amy Rapkiewicz, Amit Saxena, Gerald Villanueva, Umut Sarpel

**Affiliations:** ^1^New York University School of Medicine, 550 1st Avenue, New York, NY 10016, USA; ^2^Department of Rhemutology, New York University School of Medicine, 246 E. 20 St. 1st Floor, New York, NY 10003, USA; ^3^Department of Pathology, New York University School of Medicine, 462 1st Avenue 4W39, New York, NY 10016, USA; ^4^Department of Gastroenterology, New York University School of Medicine, 550 1st Avenue, New York, NY 10016, USA; ^5^Department of Surgery, New York University School of Medicine, 550 1st Avenue, NBV15S11, New York, NY 10016, USA

## Abstract

*Background*. The hepatitis C virus is a major cause of hepatocellular carcinoma. Extrahepatic manifestations of hepatitis C include mixed cryoglobulinemia which can result in ischemic damage to multiple organs. The management of these sequelae in posthepatectomy patients is unclear. *Case Report*. A 49-year-old male with hepatitis C was found to have a 4 cm hepatocellular carcinoma on surveillance imaging. He underwent portal vein embolization followed by hepatectomy. His postoperative course was complicated by the development of splenic infarcts, small bowel ischemia, skin lesions, and liver damage. Findings of elevated cryocrit and elevated rheumatoid factor suggested the diagnosis of cryoglobulin-related vasculitis. The patient improved on supportive care. *Conclusion*. Cryoglobulinemia is associated with hepatitis C and may complicate the care of this patient population. The treatment of cryoglobulinemia posthepatectomy patients is complicated by concerns over how medications may affect the regenerating liver. Steroids should be used with caution in this setting. *Summary*. Brief report of hepatectomy complicated by vasculitis in the context of hepatocellular carcinoma secondary to hepatitis C addresses the management of mixed cryoglobulinemia in post-hepatectomy patients.

## 1. Background

Hepatitis C is the second most common viral infection globally [1], and its role in hepatocellular carcinoma is well established. Mixed cryoglobulinaemia is considered an extrahepatic manifestation of chronic hepatitis C. While its prevalence is high in patients with chronic hepatitis C [2], post-hepatectomy vasculitis flares are not well understood.

## 2. Case Report

**Figure 1:**
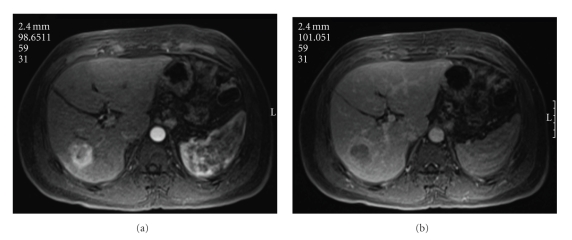
Magnetic Resonance Imaging with arterial and venous phases demonstrating a mass in segment 6 consistent with hepatocellular carcinoma.

A 49-year-old Guinean male was diagnosed with hepatitis C, genotype II in 2007. He was given a 6-month trial of ribavirin and interferon without improvement in his viral load. Recently, the patient was found to have a liver mass on screening magnetic resonance imaging. The 4 cm mass was located in segment 6 and demonstrated hyperattenuation on arterial phase and hypoattenuation on venous phase, consistent with hepatocellular carcinoma (Figure 1). The patient's past medical history is significant for hypertension and history of intermittent lower extremity skin lesions. previous biopsy of these skin lesions demonstrated leukocytoclastic vasculitis.

When evaluated by the surgical oncology service, the patient reported a recent weight loss of 10 lbs, occasional fevers, and decreased appetite. Physical examination was unremarkable, and there were no signs of ascites, malnutrition, or hepatomegaly. Liver transaminases were mildly elevated (aspartate aminotransferase 70 U/L, alanine transaminase 89 U/L) with a normal total bilirubin (0.7 mg/dL). Platelet count was 271 × 10^9^/L, and albumin and coagulation factors were within normal limits. 

In preparation for surgical resection, the patient underwent a right portal vein embolization with coils in order to hypertrophy the liver remnant. The patient was discharged the following day in a stable condition. Five weeks following the right portal vein embolization the patient underwent a computed tomography scan that demonstrated significant hypertrophy of the left lobe.Subsequently, the patient underwent an uneventful right hepatectomy. The patient tolerated the procedure well and did not require blood transfusions. His postoperative course was uncomplicated, and he was discharged home on postoperative day five at which point his liver enzymes and bilirubin had returned to their preoperative baseline. Pathologic evaluation of the specimen confirmed the presence of a 3.5 cm hepatocellular carcinoma without vascular invasion. In addition, evaluation of the gallbladder revealed incidental histologic evidence of leukocytoclastic vasculitis.

**Figure 2:**
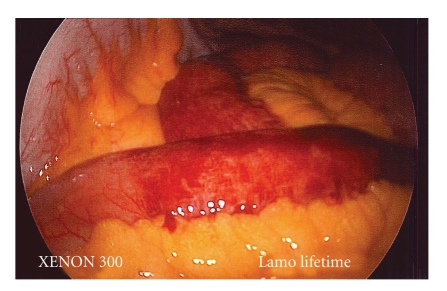
Emergent exploratory laparoscopy findings of patchy hemorrhagic serositis of the small intestine with adjacent areas of normal bowel.

On postoperative day 10, the patient presented to the emergency department with abdominal pain, nausea, and an episode of hematemesis. He denied fevers, chills, or diarrhea. On examination, the patient was found to have right 4th fingertip erythema and edema. The patient's liver transaminases were elevated relative to discharge (aspartate aminotransferase 450 U/L, alanine transaminase 662 U/L), suggesting an acute hepatic insult. A computed tomography scan demonstrated focal areas of small bowel thickening and splenic infarcts, which were suggestive of embolic disease. The patient underwent an emergent exploratory laparoscopy to rule out small bowel ischemia. Findings included diffuse patchy areas of hemorrhagic serositis along the small intestine; however, there was no evidence of bowel ischemia (Figure 2) and no bowel resection was performed. Cultures of the peritoneal fluid were negative for growth. 

Given the patient's presentation with four sites of ischemic injury (small intestine, liver, spleen, skin), the diagnosis of embolic disease or systemic vasculitis was considered. Embolic work-up with transesophageal echocardiogram was negative, and computed tomography angiography revealed a patent abdominal aorta and major branch vessels without evidence of thrombus.

Given the patient's history of hepatitis C infection as well as that of skin lesions with evidence of leukocytoclastic vasculitis on biopsy, he was also evaluated for hepatitis C virus-related vasculitides. The patient's laboratory results were significant for an elevated rheumatoid factor (104 IU/m), C3 and C4 were within normal limits (112 mg/dL, 14.9 mg/dL, resp.), Anit-Nuclear antibodies and Anti-Neutrophil Cytoplasmic Antibodies were negative, urinalysis was within normal limits, and the cryocrit was ≥3% on days 1 and 5. This led to a diagnosis of hepatitis C virus-related cryoglobulinemia. The patient's abdominal pain was self-limited, and he recovered with supportive care and had normal resumption of bowel function. His digital ischemia persisted but remained stable. He was discharged home on hospital day 10. 

**Figure 3:**
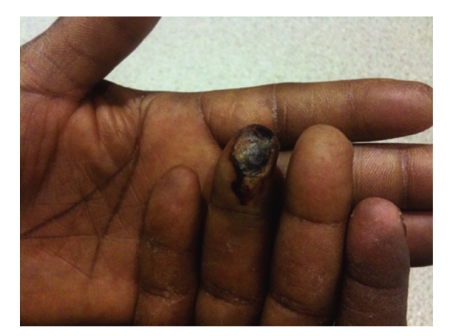
Ischemic ulceration of right fourth digit finger tip.

On outpatient follow-up, twelve days after discharge, patient's right 4th fingertip had developed into a dry area of eschar (Figure 3). Concerns regarding the progression of the vascular insult to the patient's finger lead to the initiation of therapy consisting of prednisone 30 mg once a day as well as 10 days of amoxicillin/clavulanate and aspirin. Seven weeks after discharge, the patient is improving and the digit is healing.

## 3. Discussion

Hepatitis C virus induced mixed cryoglobulinemia is one the most severe and common extrahepatic manifestations of the virus [3]. It is a systemic vasculitis resulting secondary to the deposition of cryoglobulins, which are immune complexes consisting of rheumatoid factor, IgG, hepatitis C virus ribonucleic acid, and complement. The standing relationship between hepatitis C virus and mixed cryoglobulinemia has established hepatitis C virus as a vital agent in the pathogenesis of mixed cryoglobulinemia. Furthermore, the severity of mixed cryoglobulinemia is correlated with the severity of the hepatitis C [4].

Mixed cryoglobulinemia is characterized by a clinical triad of skin lesions, arthralgia, and weakness accompanied by a high serum rheumatoid factor [3]. It mainly affects small and medium sized vessels with the finding of leukocyctolcastic vasculitis, which is the histological hallmark of mixed cryoglobulinemia [5]. The most commonly involved organ is the skin with more than 90% of patients presenting with intermittent skin lesions beginning in the lower extremities [5]. In retrospect, the diagnosis of hepatitis C virus-associated mixed cryoglobulinemia is consistent with the patient's past dermatologic history. Other clinical manifestation of hepatitis C virus-induced mixed cryoglobulinemia includes the progression of the purpuric lesions to chronic ulcers and gangrene which could represent the underlying etiology of the patient's right 4th digit findings [3]. 

Vasculitis involving multiple organs including the gastrointestinal tract is unusual. In a retrospective study of 49 patients with mixed cryoglobulinemia, the involvement of the gastrointestinal tract presenting as abdominal pain was described in 14% of the patients [6]. 

There is no diagnostic criterion for mixed cryoglobulinemia; however serologic testing for cryoglobulins is necessary to determine the type of mixed cryoglobulinemia. While cryoglobulins can be detected in 40%–60% of patients with hepatitis C virus, cryoglobulinemia vasculitis develops in only 5%–10% of those patients [7]. The presence of the cryoglobulinemia plays a vital role in establishing the diagnosis, however, the severity of the condition does not correlate to the serum levels [5]. 

Even though there is no consensus on the optimum treatment for mixed cryoglobulinemia, given the central role that hepatitis C virus plays in the pathogenesis of mixed cryoglobulinemia, the treatment of the underlying viral infection is essential. Due to the potential side effects, treatment with ribavirin and alpha-interferon should only be established after reviewing any contraindications [5]. The treatment duration currently suggested is 12 months with Peg-Interferon and ribavirin [7]. Immunosuppressive agents, normally a combination of corticosteroids, cyclophosphamide, and azathioprine, are reserved for patients with severe disease [7]. Low dose corticosteroids are used to manage minor manifestations of vasculitis but do not play a role in severe cases with multiple organ involvement [7]. Case studies have shown plasmapheresis to be safe and that it may play a role in the management of glomerulonephritis, neuropathy, and purpura [3]. Rituximab is a promising new treatment for MC as demonstrated by several studies. The overall prognosis of mixed cryoglobulinemia is quite unfavorable with the 10-year mean survival of only 50%–60% [5].

The risk of steroid treatment in the immediate post-hepatectomy period is unclear, and no human studies in the literature address this issue. There are two areas of concern: first, steroid therapy may directly inhibit liver regeneration and second, steroid-induced immunosuppression may lead to a flare of the underlying hepatitis. Whether steroids retard liver regeneration in humans is unstudied, however in animal models of hepatectomy, steroids have been shown to affect the regeneration of the liver remnant [8]. Rats who were subjected to 70% hepatectomy and then started on prednisone demonstrated fewer hepatic mitotic figures than the control hepatectomy group [8]. In control rats, the mitotic index was 1.77; in the rats given methylprednisolone the mitotic index was 0.28 (*P* = .01) [8]. This phenomenon may also impair regeneration of liver parenchyma in human post-hepatectomy patients which is vital for clinical recovery. 

In conclusion, hepatitis C virus-related cryoglobulinemic vasculitis is not uncommon and can manifest unexpectedly with ischemic injury to multiple organ sites. In the case of this patient, it is possible that the hepatectomy itself may have caused the flare of vasculitis. Post-operative hepatitis flares are known to occur, and since mixed cryoglobulinemia is related to the course of the hepatitis C virus, it is reasonable to suppose that the events are related. Given that hepatitis C virus is the second most common chronic viral infection, as well as the possible exacerbation of the vasculitis secondary to surgery, surgeons involved with hepatitis C patients need to be aware of this entity. Steroid therapy in the regenerative phase following hepatectomy must be used with caution since it may adversely affect hepatic regeneration.
